# The role of peer reward and punishment for public goods problems in a localized society

**DOI:** 10.1038/s41598-020-64930-4

**Published:** 2020-05-19

**Authors:** Hiroki Ozono, Yoshio Kamijo, Kazumi Shimizu

**Affiliations:** 1 Faculty of Law, Economics and Humanities, Kagoshima University, 1-21-30, Korimoto, Kagoshima, 890-0065 Japan; 2 School of Political Science and Economics, Waseda University, 1-6-1, Nishi-Waseda, Shinjuku-ku Tokyo, 169-8050 Japan

**Keywords:** Evolution, Psychology, Environmental social sciences

## Abstract

Cooperation in social dilemmas can be sustained if individuals are effectively rewarded or punished from peers within the group. However, as group size increases, we inevitably face localization, in which a global group is divided into several localized groups. In such societies, members can reward and punish only neighbors within the same localized group, while cooperation for social dilemmas should be solved through global group involvement. In this situation, the global group and the local group are not always equal in terms of welfare, and situations can arise in which cooperation is beneficial for the global group but not for the local group. We predict that in such a locally inefficient situation, peer reward and punishment cannot function to sustain global cooperation. We conducted an experiment in which 16 group members played a public goods game incorporating peer reward and punishment. We manipulated the range of peer reward and punishment (only local members/all global members) and payoff structure (locally efficient/locally inefficient). We found that high cooperation was not achieved and that peer reward and punishment did not function when, and only when, the group was divided into localized groups and the payoff structure was locally inefficient.

## Introduction

The challenge associated with fostering and maintaining cooperation within a large-scale society^1–3^ has been well-demonstrated in a public goods game (PGG). In a PGG, members of a group decide to contribute to a common project, and the total contributions are multiplied and shared equally among group members. In this situation, non-cooperators have an advantage over cooperators because they do not contribute but receive benefits from others’ contributions. Thus, a group faces a serious challenge in providing public goods. Numerous studies have suggested that peer punishment and/or reward (non-cooperators are punished or cooperators are rewarded) could solve this problem because non-cooperators would receive fewer benefits than cooperators^4–9^. For example, Rand *et al*.^9^ conducted an experiment revealing that both reward and punishment facilitate cooperation in a PGG and that participants are more likely to choose reward over punishment if they can choose either.

In this study, we examine how localization—in other words, subgrouping of the global group—influences cooperation in a PGG that has peer reward and punishment. Historically, society has been growing increasingly larger. Especially after the agrarian revolution, group size increased tremendously compared with hunter-gatherer societies because of high productivity and settlement^10^. The localization of a global group occurs inevitably when group size increases^11–14^. In a large society, the reach of reward and punishment is limited for several reasons. For instance, due to geographical barriers, it is difficult for the Japanese to reward Americans who act in an environmentally friendly way; in a slash-and-burn agricultural society, it is difficult for a villager to punish a person in another village who burns too many trees in the common forest^15^. Furthermore, because available resources that one owns are limited, this constrains the number of individuals one can reward or punish. Thus, the more people are committed to a common project, the more difficult it is to reward and/or punish other members sufficiently to provide public goods. In addition, as the population becomes larger, a new common problem sometimes occurs among local groups. For example, while all villages near a lake can enjoy its fishery resources, management of fishery resources is usually imposed on villages located along the lake’s coast.

Based on the argument above, we incorporated the localization factor with peer reward and punishment in PGG experiments. Localization occurs considerably in real society, and examining its influence is consequential in discussing to what extent peer reward and punishment contribute to solving public goods problems in a global society.

We have to separately consider the welfare of global and local groups when the global group is composed of multiple localized groups. In this paper, the welfare of a global/local group means the sum of payoffs of global/local members. By definition, global public goods should be welfare-enhancing for global members if they all contribute. However, global public goods are not necessarily welfare-enhancing for local members even if they contribute to global public goods. Hence, a resolution of the conflict between global-level and local-level welfare is essential to achieve high cooperation in public goods situations. We predict that humans are more likely to behave to increase mutual profit among local group members, with whom they can interact directly, rather than to increase the profit of global group members with whom they can interact less directly. This prediction aligns with results of previous studies. It is well documented that humans tend to cooperate more with in-group than out-group members^16–20^, and humans are likely to sympathize with people close to them (such as family members and neighbors)^21–24^. Therefore, when conflict occurs between global and local welfare, members of a localized society might not be motivated to achieve global cooperation in PGG through peer reward and punishment. Hereon, we explain our experimental settings in detail and introduce our hypotheses.

In the experiment, we prepared four conditions. First, a single group with four members, following the experimental framework of Rand *et al*.^9^, was a baseline condition. In this condition (the BASE condition), four group members decided how much to contribute to the common pool, and they decided whom to punish or reward and by how much. The other three conditions had 16 members each. In two of the three conditions, 16 members were divided into four groups of four members. In these two conditions (the Local0.4 and Local0.1 conditions, as explained below), PGGs were played with all 16 global group members; however, interaction of reward and punishment were realized only with the four same local group members. In the last condition (the Global0.1 condition), the 16 members were not divided into local groups, and all 16 played the reward and punishment interactively, as well as the PGG.

**Figure 1:**
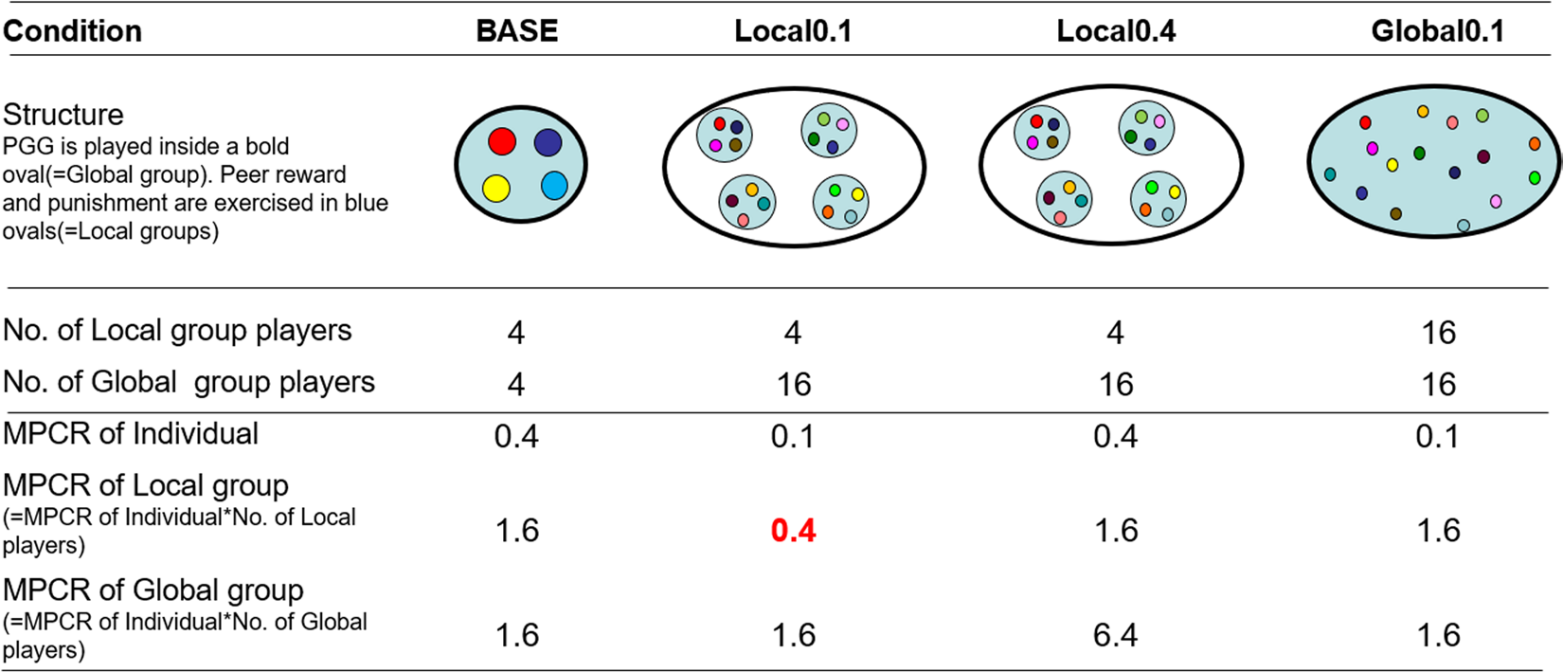
Experimental settings and MPCR for each layer.

When a global group consists of four local groups—as for conditions Local0.4 and Local0.1—we set the locally efficient and the locally inefficient condition by manipulating MPCR (Marginal Per Capita Return), ordinarily defined as marginal change of individual profit per contribution. In this paper, we term this the “MPCR of individual.” In addition, we call marginal change of local group profit per contribution the “MPCR of local group” and that of global group profit per contribution the “MPCR of global group.” For example, when 16 members participate in a PGG and are divided into 4 local groups consisting of 4 members each and the multiplication factor for the PGG is 6.4; one contribution by a member results in 0.4 (= 1*6.4/16) gain for a member (MPCR of individual), 1.6 (= 4*6.4/16) gain for a local group itself (MPCR of local group), and 6.4 (16*6.4/1.6, multiplication factor for the PGG) gain for a global group as a whole (MPCR of global group). In this way, we can clearly discuss whether cooperation in the PGG is efficient enough to increase each level’s welfare, that is, individual, local, and global levels. The critical point of being efficient and inefficient is the value 1 of the MPCR (see Fig. 1 for an illustration of the experimental settings and MPCRs).

In localized group conditions, we set the MPCR of individual to 0.4 and 0.1 and termed these Local0.4 and Local0.1, respectively. The multiplication factor for the PGG was 6.4 in Local0.4 and 1.6 in Local0.1 conditions. In both conditions, cooperation in PGG reduces individual welfare but increases global welfare, so these conditions have normal PGG payoff structures. However, these two conditions differ at the local group level. In the Local0.4 condition, the MPCR of local group is 1.6 (= 4*6.4/16), which is more than 1, meaning cooperation increases the welfare of the local group as well and, in this sense, this condition is locally efficient. However, in the Local0.1 condition, the MPCR of local group is 0.4 (= 4*1.6/16), which is below 1, and local group welfare decreases by one contribution. Thus, although cooperation is efficient on a global level, it is inefficient on a local level. In this situation, the incentive to facilitate cooperation with one’s local group members, with whom the member can interact directly, would be reduced, and reward for cooperators and punishment for non-cooperators would be less likely to occur. Consequently, cooperation would be less likely in the Local0.1 than the Local0.4 condition.

Finally, we prepared the Global0.1 condition, which had 16 group members, but they could mutually reward and punish all 16 group members. In the Global0.1 condition, the multiplication factor for the PGG was 1.6 and the MPCR of an individual was 0.1 (= 1*1.6/16), which was the same as for Local0.1. If the MPCR of an individual is the only thing that matters, it is reasonable to speculate that achieving cooperation would be equally as difficult in both Global0.1 and Local0.1 conditions because both conditions employ the same levels of MPCR of an individual. However, all members could directly interact with each other in the Global0.1 condition; therefore, there was no distinction between local and global groups, and so this was the same as the BASE condition. The MPCR of local (=global) group was 1.6 (= 16*1.6/16) in the Global0.1 condition, and cooperation was efficient for increasing local (=global) group welfare. Therefore, we predicted that peer reward and punishment power to facilitate cooperation would be the same for Global0.1 as for BASE and Local0.4 conditions. As a whole, we predicted that PGG cooperation was more likely to be achieved by peer reward and punishment when group cooperation was locally efficient (Local0.4, Global0.1, and BASE) than in the locally inefficient (Local0.1) condition.

Three hypotheses (H1–H3) were derived based on the arguments above. First, we predicted that PGG cooperation was less likely to be achieved in the Local0.1 condition.

H1: PGG contribution is lower in the Local0.1 than the other three conditions.

Hypotheses H2 and H3 concern reward and punishment behaviors. To facilitate PGG cooperation, cooperators should be rewarded more than non-cooperators, and non-cooperators should be punished more than cooperators. The linkage between reward/punishment and PGG cooperation level would be weaker in the Local0.1 than the other three conditions.

H2: The positive linkage between the PGG cooperation level and received reward is weaker in the Local0.1 than the other three conditions.

H3: The negative linkage between the PGG cooperation level and received punishment is weaker in the Local0.1 than in the other three conditions.

## Results

**Figure 2:**
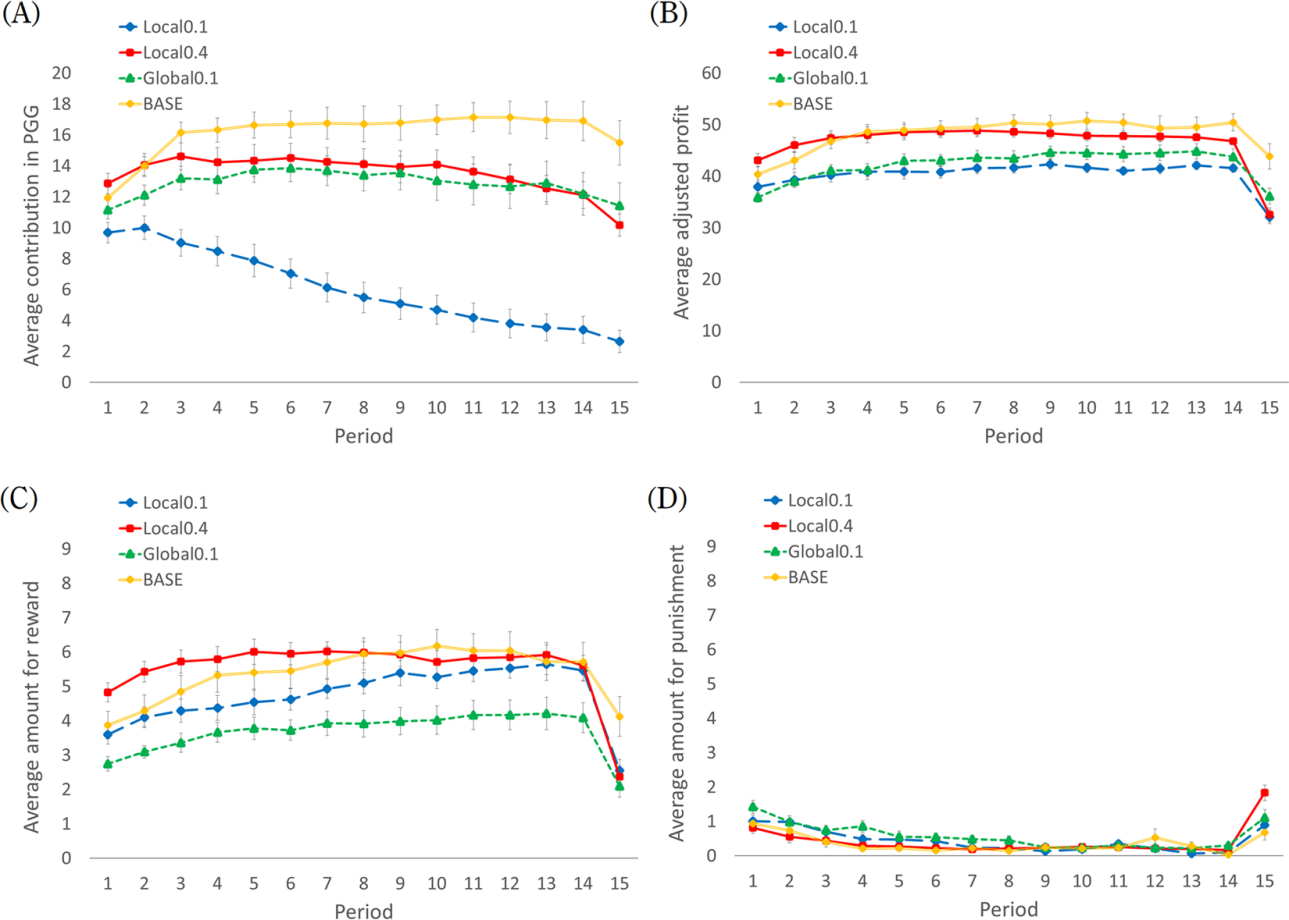
Average contribution to the public goods (**A**), average profit after adjustment (**B**), average amount for reward (**C**), and average amount for punishment (**D**) over 15 periods of play under BASE (*n* = 18), Local0.1 (*n* = 10), Local0.4 (*n* = 10), and Global0.1 (*n* = 12) conditions. Error bars indicate standard errors. Average profit was adjusted for Local0.4 because the MPCR of the global group was 6.4 only in the Local0.4, and PGG contributions produced more profit than the other three conditions, so direct comparison was difficult. To address this problem, we calculated players’ profit in the Local0.4 condition by replacing the MPCR of the global group of 6.4 with 1.6, which was equal to the other three conditions. In this way, we could directly compare profit among the four conditions.

The average PGG contribution, average amounts of reward and punishment, and average profit of players were calculated for each period (Fig. 2). First, we analyzed group-level data. Mann–Whitney U-test with Bonferroni’s correction was conducted to determine whether a difference existed between conditions. All indexes were calculated for 14 periods but not for the last (15th) period because all participants knew that this period was the last—clearly, reward decreased and punishment increased in this period. This tendency was consistent with a previous study^25^. Although this phenomenon is interesting, it is outside the scope of our research. Concerning the average PGG contribution, members under the Local0.1 condition contributed less to the PGG pool than under Local0.4 (*Z* = 3.477), Global0.1 (*Z* = 3.429), and BASE conditions (*Z* = 4.075, all *P* < 0.001). There were no significant differences among Local0.4, Global0.1, and BASE conditions (all *P* > 0.10). In addition, we divided data into the first (periods 1–7) and second (periods 8–14) halves and then calculated each average contribution. Wilcoxon’s signed-rank tests were used to analyze differences between the first and second halves for each condition. A decline of contribution occurred only in the Local0.1 condition (*Z* = 2.803, *P* = 0.005); however, there were no differences in Local0.4 and Global0.1 conditions (*Z* = 1.376, *P* = 0.169; *Z* = 0.235, *P* = 0.814, respectively), and an increase of contribution in the BASE condition (*Z* = 1.960, *P* = 0.050). Results were consistent with H1.

Concerning average profit, members under Local0.4 and BASE conditions obtained more profit than did Local0.1 and Global0.1 (Local0.4 vs Local0.1, *Z* = 3.099, *P* = 0.006; Local0.4 vs Global0.1, *Z* = 2.638, *P* = 0.042; BASE vs Local0.1, *Z* = 3.212, *P* = 0.006; and BASE vs Global0.1, *Z* = 3.006, *P* = 0.012). Regarding reward, members under Global0.1 rewarded others less than under Local0.4 (*Z* = 3.298, *P* < 0.001) and BASE conditions (*Z* = 2.582, *P* = 0.054). There were no significant differences in punishment among conditions (all *P* > 0.10). In addition, amounts of reward were greater than amounts of punishment for all four conditions (all *P* < 0.001), consistent with results of Rand *et al*.^9^, thus participants chose reward rather than punishment.

**Figure 3:**
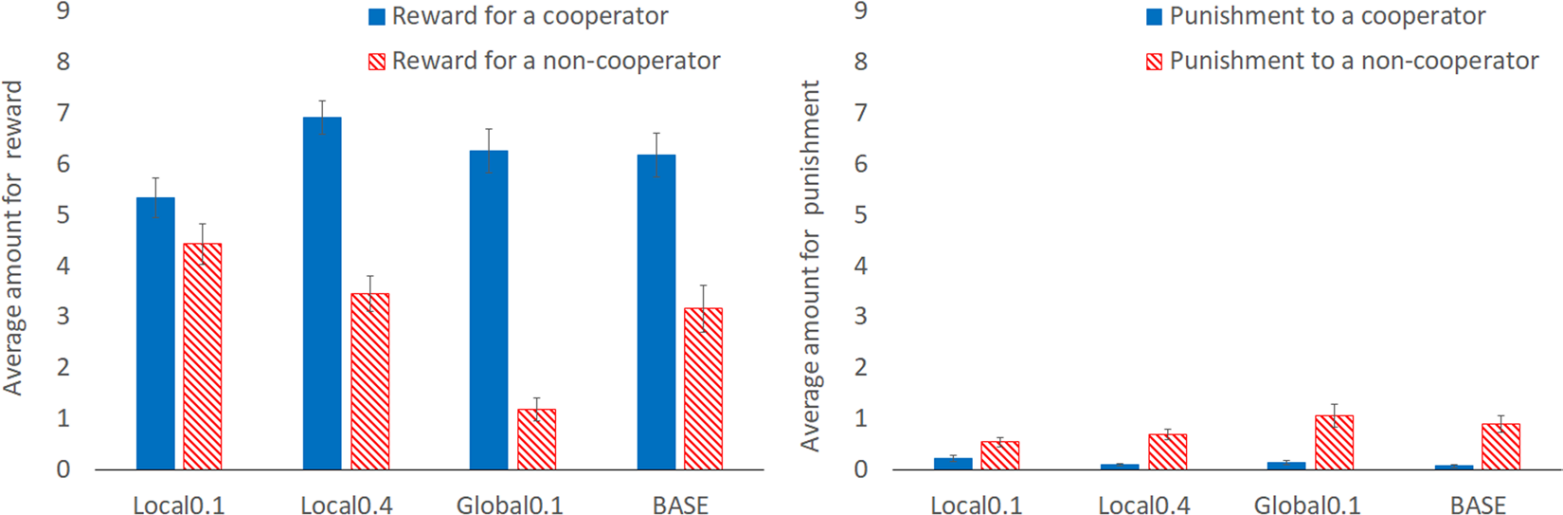
Average amount of reward and punishment to cooperator (equal-to- or above-average contributor)/non-cooperator (below-average contributor) over periods 1–14. Error bars indicate standard errors.

Next, we analyzed reward and punishment behaviors in detail. Figure 3 shows the average amount of reward and punishment to one cooperator, who contributed equal-to-average or above-average in PGG, and to one non-cooperator, who contributed below-average in PGG, over periods 1–14. In all conditions, cooperators were rewarded more than non-cooperators, and non-cooperators were punished more than cooperators. In other words, there were linkages between PGG cooperation and reward/punishment behaviors. Thus, reward and punishment might function to facilitate cooperation in the PGG. However, this tendency was weaker for reward in the Local0.1 than the three other conditions. To test this statistical significance, the difference between reward for a cooperator and that for a non-cooperator was calculated and analyzed using the Mann–Whitney U-test with Bonferroni’s correction. The difference in reward between a cooperator and non-cooperator was smaller in the Local0.1 than in Local0.4 (*Z* = 3.175, *P* = 0.006), Global0.1 (*Z* = 3.824, *P* < 0.001), and BASE conditions (*Z* = 2.733, *P* = 0.030), meaning that reward to PGG cooperators was lower in Local0.1 than in the other three conditions, and so H2 was supported. In addition, the difference in reward was larger in the Global0.1 than the BASE condition (*Z* = 2.794, *P* = 0.024). We analyzed for punishment in the same manner; however, there were no significant differences (all *P* > 0.10) and so H3 was not supported.

We further investigated H2 and H3, using multilevel regression analyses with individual data. Results were consistent with the group-level analysis reported above. In all conditions, we found a linkage between received reward and PGG cooperation level, and this linkage was weaker in Local0.1. Regarding punishment, the difference only in Local0.1 was unclear (see SI Appendix, section 1 for details). In addition, we examined how players changed their cooperation level in PGG after receiving reward and punishment in the previous period—that is, the effectiveness of reward and punishment in facilitating cooperation—using multilevel regression analysis. The results indicated that both reward and punishment functioned to change others’ behavior to cooperation; however, this function was weaker in Local0.1 than in the other three conditions (see SI Appendix, section 2 for details).

## Discussion

The PGG cooperation was lower in the Local0.1 than the other three conditions, consistent with H1. This might be partly because reward behaviors differed in the Local0.1 compared to the other three conditions. The results of reward were consistent with H2, meaning that the positive linkage between the PGG cooperation level and the received reward was weaker in the Local0.1 than in the other three. In the Local0.1 condition, reward was used not for facilitation of PGG cooperation but rather for mutual benefit within local group members. It is important to consider why results were inconsistent only for punishment, H3. This is partly because punishment behaviors were rare (Figs. 2D and 3), and detecting statistical differences among conditions for punishment might be difficult. Further analyses in the supplementary analysis 2 (SI Appendix, section 2) suggest that reward and punishment had a lower function in changing others’ behavior to cooperation in the Local0.1 than in the other three conditions. This might be because changing behaviors to cooperation decreased local-level and individual-level welfare in the Local0.1, and the members had no compelling reason to cooperate even when they were rewarded or punished.

Total profits were lower for Local0.1 than Local0.4 and BASE conditions because of lower cooperation in PGG. There was no significant difference between Local0.1 and Global0.1 conditions. This is because reward was lower in Global0.1 than in the other three conditions (Fig. 2C); participants in the Global0.1 could not gain as much profit from reward as in Local0.4 and BASE conditions. In the Global0.1 condition, it was more difficult to choose whom to reward due to the group’s number of members, so participants might have refrained from rewarding others. Nonetheless, cooperation in Global0.1 was just as high as in Local0.4. This means that under the Global0.1 condition the participants were rewarded much less but cooperation was sustained. This phenomenon should be explained. We suggest that the reward effect might have been stronger in Global0.1 than in the other three. As we can see in Fig. 3, the difference in reward between a cooperator and non-cooperator was apparently larger in Global0.1 than the other three. This means that participants in Global0.1 distinguished cooperators and non-cooperators more strictly than the participants in other conditions. This might be because the participants in Global0.1 could reward the other 15 members while the participants in the other three conditions could reward only 3 other members. Therefore, the participants could select those individuals who should be rewarded more severely in Global0.1, which resulted in a larger distinction between cooperators and non-cooperators. This “severely distinguished reward” might lead to a stronger effect to facilitate cooperation, which compensated for the lower level of reward in Global0.1.

The results of our study, which is consistent with H1 and H2, implied that humans can consider and facilitate only local group welfare. We need global cooperation beyond the local level in our modern society, and the slogan “think globally, act locally” is all too familiar. However, our results suggest a difficulty of global cooperation beyond local communities and imply that we can only “think locally, act locally”—we might be able to consider only local-level profit and reward to facilitate cooperation only for our local group. This understanding of humanity is consistent with the claim that we humans care about, sympathize, and cooperate only with in-group or local group members, by recent evolutionary-minded social scientists^26^.

In our real large-scale society, a situation such as Local0.1 seems to be more common than situations such as Global0.1 or Local0.4. We discussed localization’s frequent occurrence in large-scale society earlier in this paper. In addition, we believe that the decline of MPCR (of individual) frequently occurs in large-scale society. There are examples of a wide variety of declining trends, including environmentally friendly behaviors, use of public transport to decrease traffic jams, and labor to maintain irrigation systems. In these examples, cooperation by one member has less impact on other members as group size increases. In contrast, situations in which MPCR does not change regardless of group size are very rare, for instance, pure public goods such as defense costs. Thus, many PGG situations in large-scale groups inevitably face both localization and low MPCR, as in the Local0.1 condition, and this might result in low cooperation levels. Therefore, high cooperation by peer reward and punishment might be limited to small-scale societies such as families, some hunter-gatherers, and some local communities. In larger-scale societies, it can be effective to establish systems other than peer reward and punishment. The pool punishment system and/or the leader support system are important candidates for solving the problem^27–32^. The limitations of peer reward and punishment emphasize the importance of investigation into how these systems emerge and are maintained.

We should note, however, that it would be too hasty to induce a general conclusion that says “humans can consider and facilitate only local group welfare” or “humans can only think locally, act locally” from our experimental results. We suggest that there were limitations to our study and we propose future research directions. First, the sample size was too small at a group level (10 to 18 groups in each condition); therefore, caution must be exercised when generalizing the results of the present study. This is especially true given the fact that the participants for this study all came from a single culture: Japanese. Larger scale studies, incorporating people of diverse backgrounds, are warranted.

Second, the payoff structure and group structure applied in our study was only one combination among the various kinds of structures, and it is unclear whether the tendency we found in the experiment could be observed in the other combinations. Capraro and Barcelo^33^ examines the public goods problem with a non-linear payoff structure. As a non-linear payoff might be more popular than linear payoff in our real-life public goods, it would be intriguing to investigate how a nonlinear payoff structure affects in the localized societies. As for group size, we examined only four or 16 group members, and it is necessary to investigate other group sizes. We used four members as a baseline because it was the most popular size in the PGG studies^34^ and 16 members (four localized groups consisting of four members each) as the larger group size in order to compare global welfare with local ones. Though 16 was the minimum group size to compare these welfares, it is not really a “larger-scale” society. A group size of around 150 might be a limitation for humanity to sustain mutual interactions, as Dunbar^12^ argued, and cooperation with more than 150 members might be difficult even in a locally efficient situation.

In addition, we were only able to examine one social structure with a hierarchy of global and local levels. However, there are other structures such as a three-level hierarchy or networks^35–37^. How these structures affect PGG cooperation with peer reward and punishment need further investigation. Related to network structure, Hauser *et al*.^38^ conducted an online experiment in which large-group members played the PGG with all group members, and then played a Prisoner’s dilemma (PD) game with a partner/partners that comprised one or two members connected in a given network. Though the MPCR of their study was so low that PGG cooperation was locally inefficient for PD pairs, they observed that the high cooperation level in the PGG and the behavior of the PD contingent on PGG cooperation became the driving force of large-group cooperation. Specifying the reason why our experiment and Hauser’s lead to different results is difficult because of the many differences between their experimental settings and ours, for instance, online vs laboratory experiments, PD vs reward/punishment, pairs vs four group members, and differing feedback information for the PGG. Future investigations to detect these reasons would help in understanding how global cooperation can be achieved only by peer interactions. Furthermore, some evolutionary dynamic simulations^39–41^ shed light on an institutional aspect of localization that our study does not consider: Localized institutions or polycentric governance are more effective in establishing cooperation than global or monocentric ones. These studies will be helpful in clarifying the impact of different types of reward and punishment systems on global commons in localized society.

Finally, we should consider alternative explanations for our results other than the “think locally, act locally” explanation. For example, as argued above, a “severely distinguished reward” is likely to occur under the Global0.1 condition, which might result in the facilitation of cooperation in PGG. Although these explanations can be complementary, we should investigate whether such an alternative or the “think locally, act locally” explanation is more plausible or whether both are equally plausible in future studies.

Our results may suggest that we humans consider only local group welfare and have difficulty in acting for a global one, and thus the function of peer reward and punishment for welfare enhancement might be limited to relatively small societies. To realize “think globally, act locally” in a globalized society, we will need an appropriate setting, where the incentive structure, group structure, and reward-punishment system are well constellated.

## Methods

The Waseda University Ethical Review Board approved this study (IRB number is 2016-007). It was conducted in accordance with approved guidelines. Written informed consent was obtained from all participants prior to beginning the experiment.

## Participants

In total, 584 university students participated in this experiment: 72 (18 groups) participated in the BASE condition, 160 (10 groups) in the Local0.1, 192 (12 groups) in the Global0.1, and 160 (10 groups) in the Local0.4. Participants were recruited via a university portal website, and monetary reward was emphasized during recruitment.

## Procedure

In all conditions, participants were assigned to laboratory booths to ensure their anonymous and independent decisions. Sixteen participants participated in each session of the experiment of Local0.1, Local0.4, and Global0.1 conditions. For the BASE condition, 8, 12, or 16 participants took part and were allocated randomly to one of the two, three, or four 4-person groups. After reading explanations on PowerPoint slides, participants completed confirmation tests concerning their understanding of the experiment’s details. Neutral words were selected for explanation. After confirming that all participants understood the experimental details, we ran one trial period and then participants started the real session.

Details of the experimental transactions are as follows. The transactions comprised two stages: PGG and reward/punishment stages. Participants were told before the experiment began that these periods would be repeated 15 times and that tokens they earned during transactions would be redeemed as remuneration.

### PGG stage

For all conditions, each member was given 20 tokens at the beginning of the stage and simultaneously chose his/her contribution from 0 to 20 in increments of 1, which were then subtracted from his/her endowment of 20 tokens. The total tokens that each member contributed were multiplied by 1.6 in BASE, Local0.1, and Global0.1 conditions and distributed equally to all four or 16 group members. In the Local0.4 condition, the total tokens were multiplied by 6.4 and distributed equally to the 16 group members. After all decisions were made, players received feedback on other players’ contributions.

### Reward/punishment stage

For all conditions, each player was given nine tokens and they simultaneously decided how many tokens to use to reward and punish other players. Impact-to-cost ratio is 3:1—if member A uses one token to reward member B, A loses one, and B gets three tokens; if C uses one token to punish D, C loses one, and D loses three tokens. Each member can use up to nine tokens to reward or punish other members, and each player keeps tokens they do not use. A participant cannot simultaneously both reward and punish the same member. When participants decide on rewards and punishments, they can refer to the previous results of both Stages 1 and 2. After all participants decide on rewards and punishments, they receive feedback concerning from which other participants they received punishment/reward and the relevant amounts.

It is noteworthy that under Local0.1 and Local0.4 conditions, participants could reward and punish only three members of the same local group; in Global0.1 and BASE conditions, participants could reward/punish all members except themselves (see SI Appendix, section 3 for details).

These two stages were repeated 15 times. We used z-Tree software^42^ to conduct experiments. Each session took an average of approximately 80 minutes. The total attained score was converted to money using the rate of 1 token = 1.5 yen (100 yen is approximately 1 US dollar) in Local0.1, Global0.1, and BASE and 0.5 yen in the Local0.4 condition. In addition, an 800-yen show-up fee was given to participants who completed the experiment. Average remuneration was 1671 yen.

## Supplementary information

Supplementary information.

Supplementary information2.

## Data Availability

All data generated or analyzed during this study are included in the Supplementary Information files.
