# Supplementary material for: The role of peer reward and punishment for public goods problems in a localized society
Source: Sci Rep. 2020 May 19;10:8211. doi: 10.1038/s41598-020-64930-4 (PMC7237688; doi:10.1038/s41598-020-64930-4)
Supplement: Supplementary file 1 — Supplementary information. [file 41598_2020_64930_MOESM1_ESM.docx]

Supplementary Information for

The role of peer reward and punishment for public goods problems in a localized society

Hiroki Ozono, Yoshio Kamijo, and Kazumi Shimizu

Hiroki Ozono

Email: hiroki.ozono@gmail.com

**This PDF file includes:**

Supplementary text

Tables S1 to S3

**Other supplementary materials for this manuscript include the following:**

Datasets S1

1. **Supplementary analysis 1**

We further investigated Hypotheses 2 and 3 (H2 and H3)—linkage between public goods game (PGG) cooperation level and received reward/punishment—by using repeated-measures data on individuals clustered within groups. Individual data over 15 periods are nested in a local group and the local group is nested in the global group for Local0.1 and Local0.4 conditions. Regarding Global0.1 and BASE conditions, the individual is nested in the local (=global) group. We excluded the final (15th) period from the data, because all participants knew it was the last and, therefore, were expected to behave differently. Thus, individual variation is relative not only to differences in conditions but also among (local and/or global) groups and periods. To examine factors motivating observed reward, punishment, and cooperation behavior more closely, we constructed various multi-level regression models, which considered random intercepts for each group and individual.

Table S1 indicates determinants of received reward. In all conditions, deviations from the group average contribution show a strong positive influence to reward. This indicates a linkage between received reward and PGG cooperation level. Interaction coefficients of Models 5, 6, and 7 indicate that this linkage is weaker in Local0.1 than in the other three conditions. These results are consistent with H2.

Table S2 indicates determinants of received punishment. In all conditions, deviations from the group average contribution show a strong negative influence to punish, and there is a negative linkage between received punishment and PGG cooperation level. Unlike the analysis of received reward, interaction coefficients of Models 5, 6, and 7 indicate that this linkage is weaker not only in Local0.1 but also in Local0.4 compared with the other two conditions. These results are not consistent with H3, but are consistent with the group level analysis (see Fig. 3 and related analysis in the manuscript).

Table S1. Determinants of received reward. Multi-level regression coefﬁcients of determinants for received reward in four conditions. Models 1, 2, 3, and 4 show analysis for each condition. Models 5, 6, and 7 compare Local0.1 with the other three conditions, respectively, allowing assessment of reward behavior between conditions. The benchmark condition for dummy variables is Local0.1. Numbers in parentheses indicate standard errors.

Table S2. Determinants of received punishment. Multi-level regression coefﬁcients of determinants of received reward in four conditions. Models 1, 2, 3, and 4 show analysis for each condition. Models 5, 6, and 7 compare the Local0.1 with and the other three conditions, respectively, allowing assessment of punishment behavior between conditions. The benchmark condition for dummy variables is Local0.1. Numbers in parentheses indicate standard errors.

1. **Supplementary analysis 2**

We investigated changed behavior after reward or punishment. We anticipated that members who were rewarded or punished might be less likely to change their behavior to cooperation in the Local0.1 than the Local0.4 condition because changing their behavior to contribute to the common pool decreased local-level and individual-level welfare, and there was no compelling reason to cooperate with their local group. We predicted that reward and punishment would have less impact on changing behavior of non-cooperators to cooperation in the Local0.1 than the other three conditions.

Table S3 indicates how players changed their cooperation levels in PGG after receiving reward and punishment. Models 2, 3, and 4 show that participants increased their contribution in period t after receiving reward and punishment in the period t − 1 in Local0.4, Global0.1, and BASE conditions (one exception is punishment in Local0.4). However, Model 1 indicates *no* influence of reward and punishment to change PGG contribution in the Local0.1 condition. Interaction coefficients of Models 5, 6, and 7 also support this finding; the changes in PGG cooperation after receiving reward and punishment are lower in Local0.1 than in the other three conditions, except for rewards in the BASE. These results are consistent with our prediction. We should note that we found no influence of punishment in the Local0.4 condition; however, A different influence of punishment is found when comparing Local0.1 and Local0.4 conditions. In addition, we did not find a different influence of rewards when comparing Local0.1 and BASE; however, the coefficient is higher in the BASE than the Local0.1. Thus, these results are not critical in relation to our main findings: reward and punishment had less impact on changing behavior of non-cooperators to cooperation in the Local0.1 than the other three conditions.

Table S3. How players change their cooperation level in PGG after receiving reward and punishment (Dependent Variable: difference in contribution from period t − 1 to period t). Multi-level regression coefﬁcients for change in cooperation level. Models 1, 2, 3, and 4 show the analysis for each condition. Models 5, 6, and 7 compare the Local0.1 with the other three conditions, respectively, allowing assessment of change in cooperation level between conditions. The benchmark condition for dummy variables is Local0.1. Numbers in parentheses indicate standard errors.

1. **Supplementary method**
   1. Instruction of the experiment

*After a brief verbal introduction, participants read the following instructions on the computer monitor telling them that they would take part in an experiment on decision making.*

**General Guidance**

This is an experiment about decision making. You will be paid for participating, and the amount of money you will earn depends on the decisions that you and the other participants make. At the end of today’s session, you will be paid privately and in cash for your decisions.

You will never be asked to reveal your identity to anyone during the course of the experiment. Your name will never be associated with any of your decisions.

At this time, you will be given 500 yens (= 5–6 dollars) for coming on time. All the money that you earn after this experiment will be yours to keep.

**Earnings**

In this experiment, you are in a group of size 16 (you plus 15 others) (*size 4 (you plus 3 others) in BASE condition) and you will be asked to make a series of choices about how to allocate a set of tokens. You *will not* be able to know each other’s identities. The group members will remain the same throughout the experiment.

The details of the experimental transactions are as follows. 16 members named A to P (*4 members named A to D in BASE condition) will play the same role. The experiment comprises two stages, the 1^st^ and 2^nd^ stages. These stages will be repeated 15 times, and the tokens you earn during transactions will be redeemed as monetary remuneration.

Now, let us explain the details of each stage.

**1^st^ stage (BASE condition)**

Each of the four members contributes 20 tokens at the beginning of this stage. The members are asked to decide how many tokens to contribute to the group pool. You lose the amount you contribute to the pool, but 40% of the sum of the tokens is given to each of the 4 members, including you. Hence, the number of tokens you contribute and the sum of tokens contributed by any participant, including you, will determine the payoff you receive. Each choice that you make is similar to the following example.

**-Examples of choices you will make in the 1^st^ stage and earnings**

Example 1: Suppose that you and the other 3 members all contribute 20 tokens to a pool. You will earn:

20 (initial endowment) − 20 (the tokens you contributed)

+ 0.4 × 80 (the sum of tokens the 4 members contributed)

=32

Example 2: Suppose that you and the other 3 members all contribute nothing. You will earn:

100 (initial endowment) − 0 (the tokens you contributed)

+ 0.4 × 0 (the sum of tokens the 4 members contributed)

=20

Example 3: Suppose that you give 4 tokens and the other members contribute 5, 10, and 16 tokens each. You will earn:

20 (initial endowment) − 4 (the tokens you contributed)

+ 0.4 × 35 (the sum of tokens the 4 members contributed)

=30

**1^st^ stage (Local0.1 and Global0.1 conditions)**

Each of the sixteen members contributes 20 tokens at the beginning of this stage. The members are asked to decide how many tokens to contribute to the group pool. You lose the amount you contribute to the pool, but 10% of the sum of the tokens is given to each of the 16 members, including you. Hence, the number of tokens you contribute and the sum of tokens contributed by any participant, including you, will determine the payoff you receive. Each choice that you make is similar to the following example.

**-Examples of choices you will make in the 1^st^ stage and earnings**

Example 1: Suppose that you and the other 15 members all contribute 20 tokens to a pool. You will earn:

20 (initial endowment) − 20 (the tokens you contributed)

+ 0.1 × 320 (the sum of tokens the 16 members contributed)

=32

Example 2: Suppose that you and the other 15 members all contribute nothing. You will earn:

20 (initial endowment) − 0 (the tokens you contributed)

+ 0.1 × 0 (the sum of tokens the 16 members contributed)

=20

Example 3: Suppose that you give 5 tokens and the total contributions of other members are 195 tokens each. You will earn:

20 (initial endowment) − 5 (the tokens you contributed)

+ 0.1 × 200 (the sum of tokens the 16 members contributed)

=35

**1^st^ stage (Local0.4 condition)**

Each of the sixteen members contributes 20 tokens at the beginning of this stage. The members are asked to decide how many tokens to contribute to the group pool. You lose the amount you contribute to the pool, but 40% of the sum of the tokens is given to each of the 16 members, including you. Hence, the number of tokens you contribute and the sum of tokens contributed by any participant, including you, will determine the payoff you receive. Each choice that you make is similar to the following example.

**-Examples of choices you will make in the 1^st^ stage and earnings**

Example 1: Suppose that you and the other 15 members all contribute 20 tokens to a pool. You will earn:

20 (initial endowment) − 20 (the tokens you contributed)

+ 0.4 × 320 (the sum of tokens the 16 members contributed)

=128

Example 2: Suppose that you and the other 15 members all contribute nothing. You will earn:

20 (initial endowment) − 0 (the tokens you contributed)

+ 0.4 × 0 (the sum of tokens the 16 members contributed)

=20

Example 3: Suppose that you give 5 tokens and the total contributions of other members are 195 tokens each. You will earn:

20 (initial endowment) − 5 (the tokens you contributed)

+ 0.4 × 200 (the sum of tokens the 16 members contributed)

=95

**2^nd^ stage**

Each of the four members are given another 9 tokens at the beginning of this stage. The members including you are asked to decide how many tokens to use to increase and reduce the tokens of other members. Efficiency of increase or reduction is 3. This means that when you use 1 token to increase the tokens of member B, you loses 1 and B gets 3 tokens and when C uses 1 token to reduce the tokens of you, C loses 1 and you lose 3 tokens. you can increase or reduce other members’ tokens as long as you use up all 9 tokens. you gets the token you do not use for increase and reduction. you cannot increase and reduce the tokens of the same members at the same time. When you decide, you can refer to the previous results of Stage2 and Stage 1. After all of you decided, you get feedback on by whom you are increased/reduced and how amount.

You can increase or reduce the tokens of all other member except for yourself.(BASE and Global0.1 conditions)

You can increase or reduce the tokens of your subgroup members only. This 16 group members are divided to four subgroups. Each group consists of four members. The members of subgroup are fixed throuout the game. (Local0.1 and Local0,4 conditions)

**Examples of choices you will make in 2^nd^ stage and earnings**

Example 1: Suppose that you use 1 token for increase and 3 tokens for reduction, and the total amount the other members use for increasing your tokens is 4 and that for reducing your tokens is 2. You will earn:

9 (initial endowment) − 4 (the tokens you used for increase and reduce the others’ tokens)

+ 3×4 (the other members use for increasing your tokens) -2×2(the other members use for reducing your tokens)

=13

These two stages are repeated 15 times. The total attained score is converted to money using the rate 1 token＝0.7 yen, and the converted amount plus 500 yen (the show-up fee) is given to you at the end of this experiment.

*After this general instruction is given, all participants start the experiment after filling out a confirmation test.*

**Confirmation Test**

Before you start to make your decision, you should have answered all the questions in the paper. Read carefully through the provided information provided and write down the number of points in the paper. We will watch you solving the examples, check whether you get the right answers, and help you if you have a problem or question.

**Before the decision making**

Good, now everybody has solved the problems. If anybody has any more questions, raise your hand now. Otherwise let’s practice how to input your decision on your computer screen.

- 1. Screen shots of computer displays during the experiment.


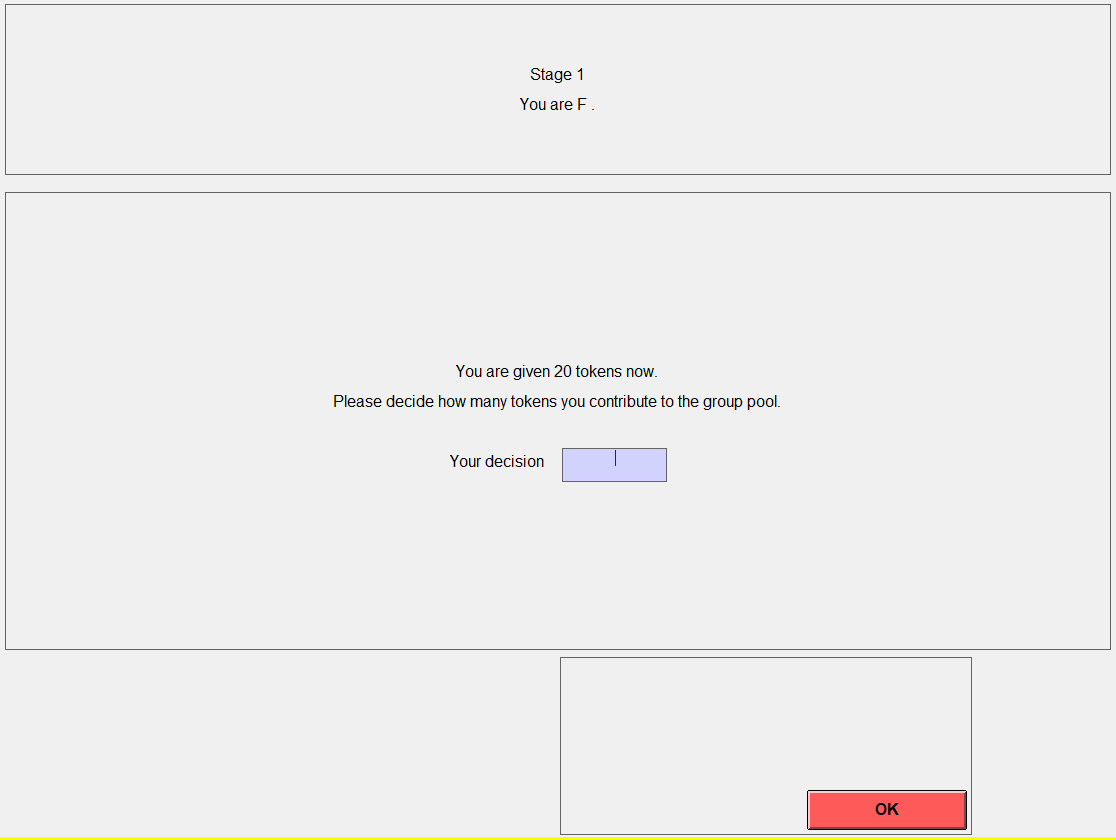


Screen shot of computer display when the participants make decisions in stage 1.


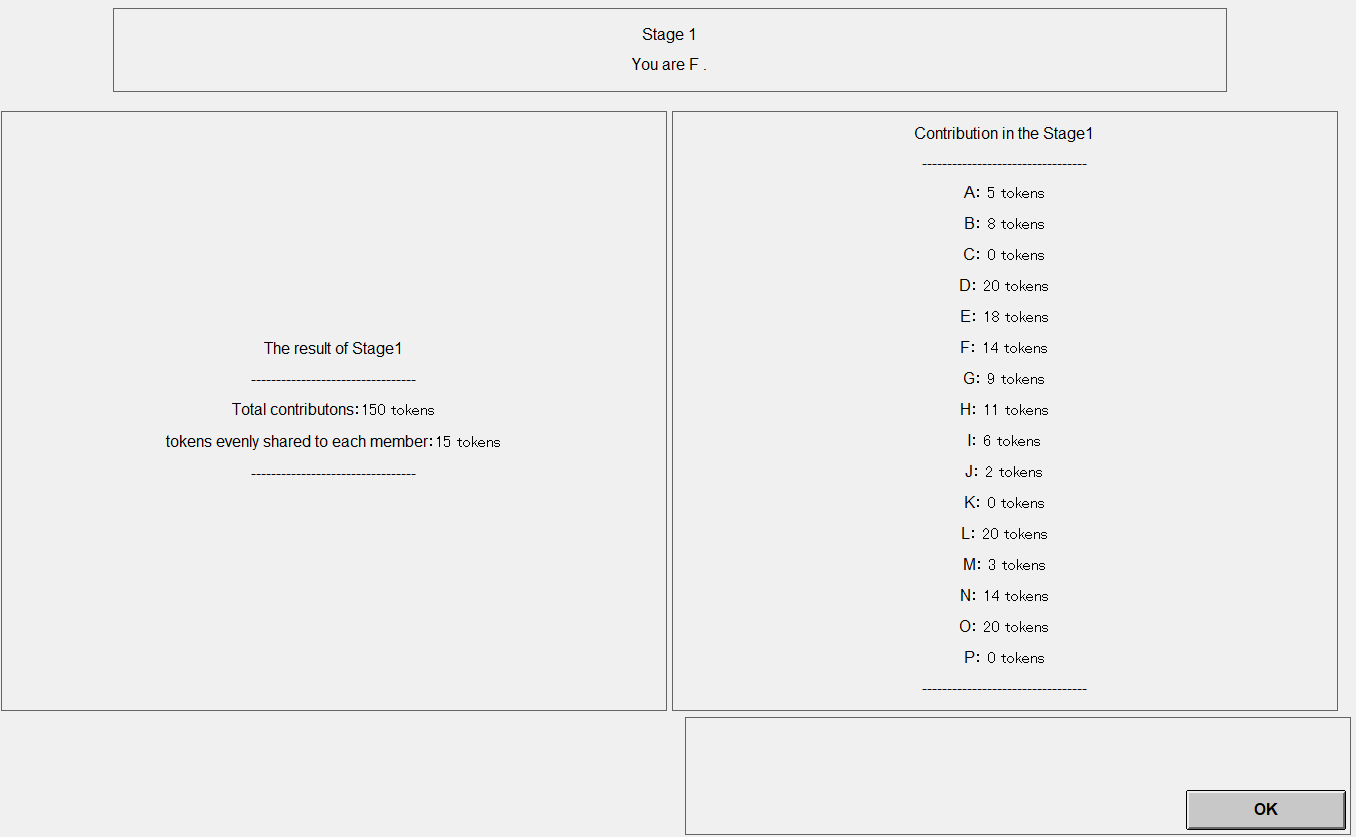


Screen shot of computer display when showing feedback after stage 1

(Local0.1 and Global0.1 conditions)


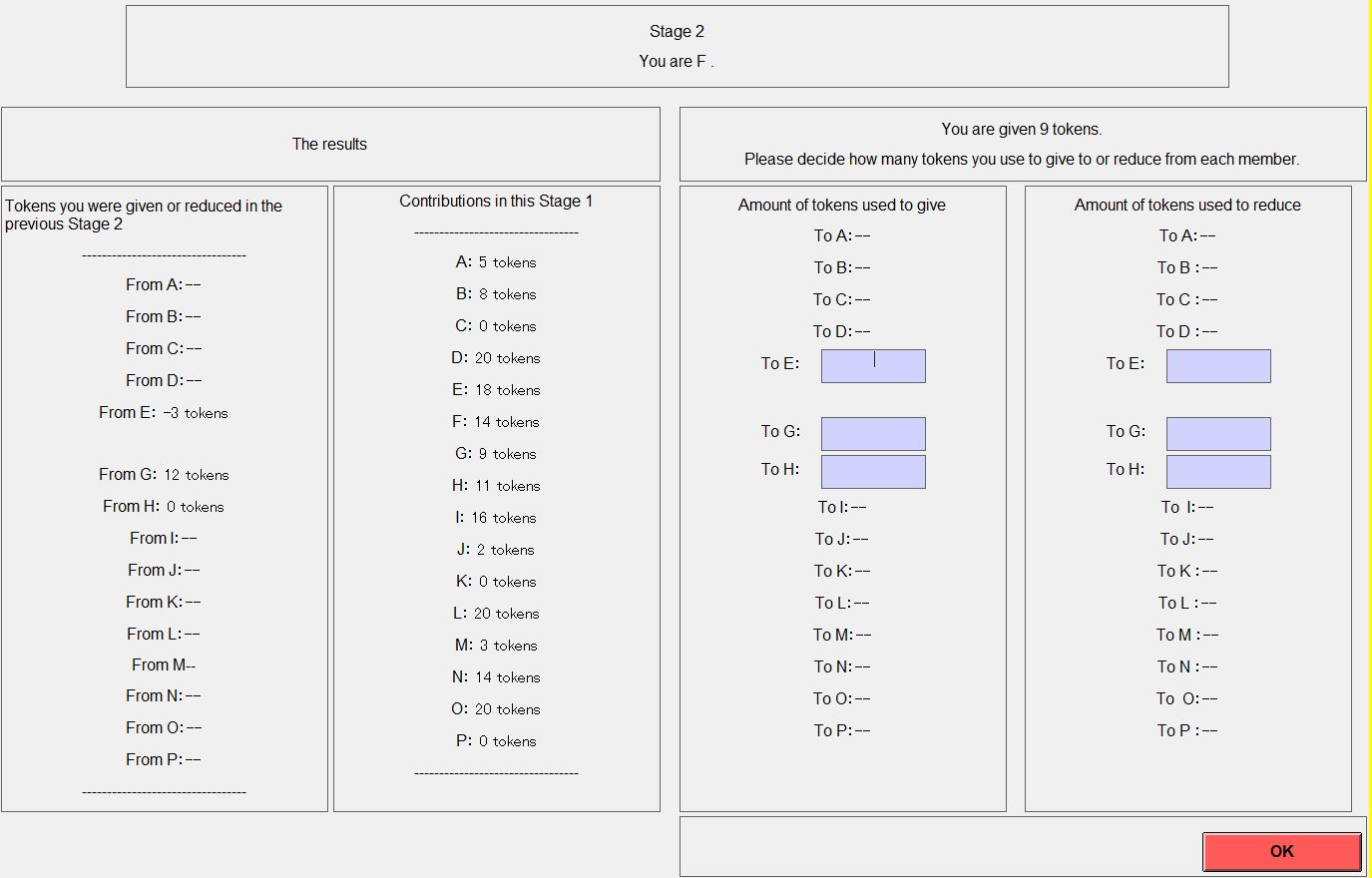


Screen shot of computer display when the participants make decisions in stage 2 (Local0.1 and Local0.4 conditions)


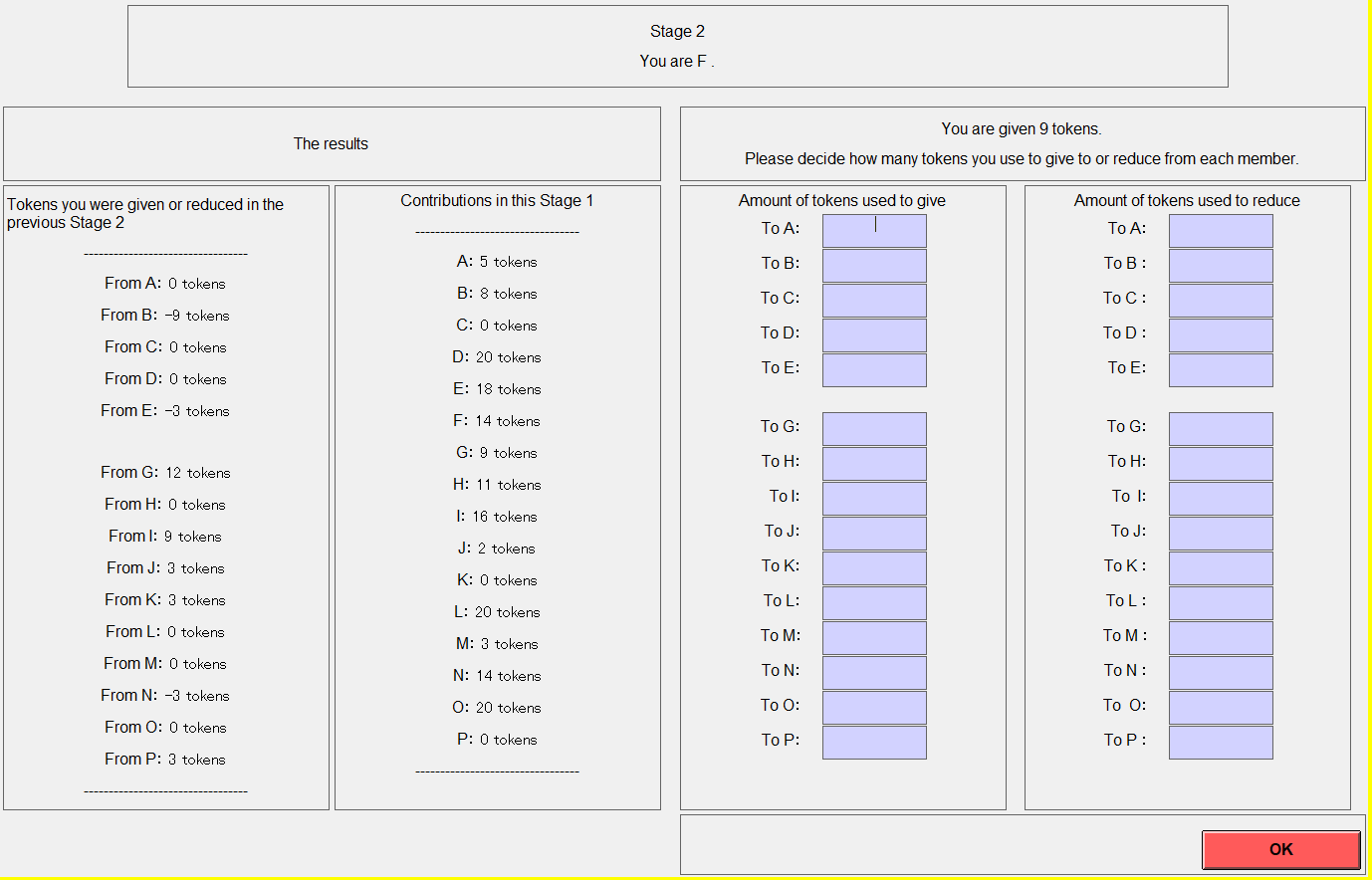


Screen shot of computer display when the participants make decisions in stage 2 (Global0.1 condition)


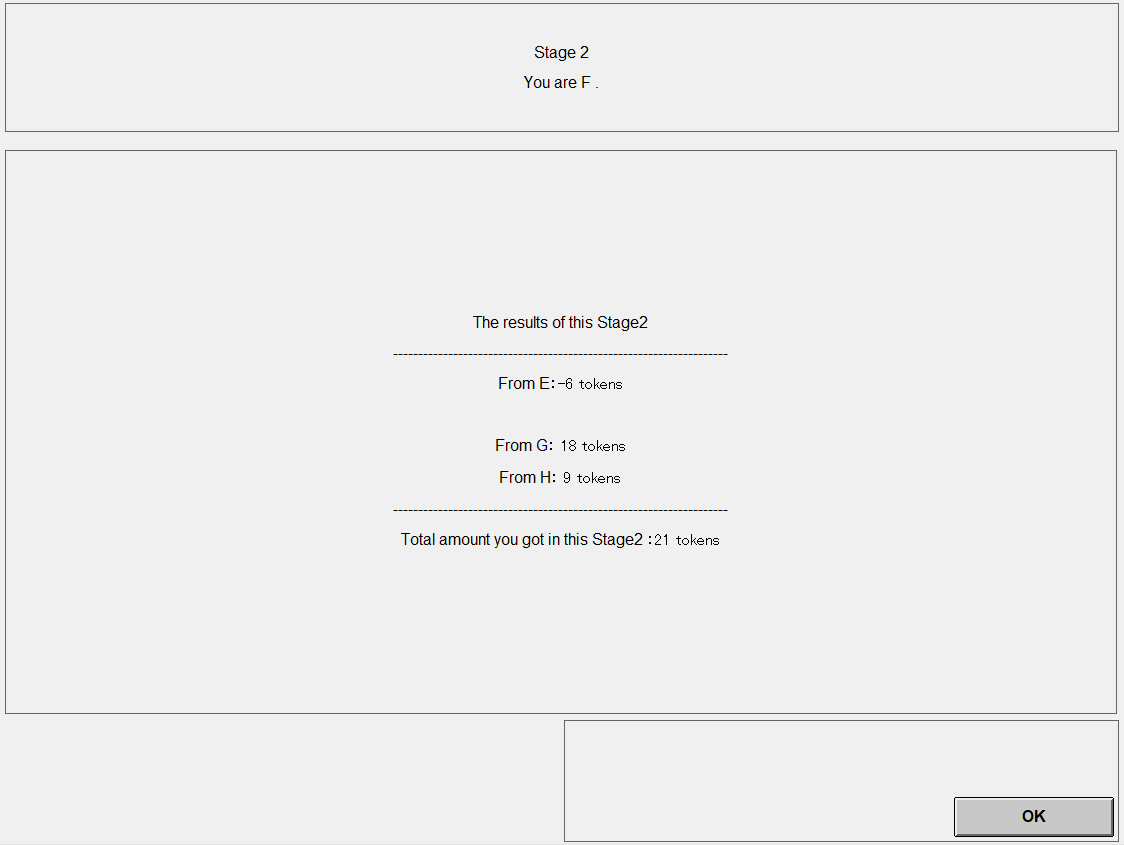


Screen shot of computer display when showing feedback after stage 2

(Local0.1 and Local0.4 conditions)


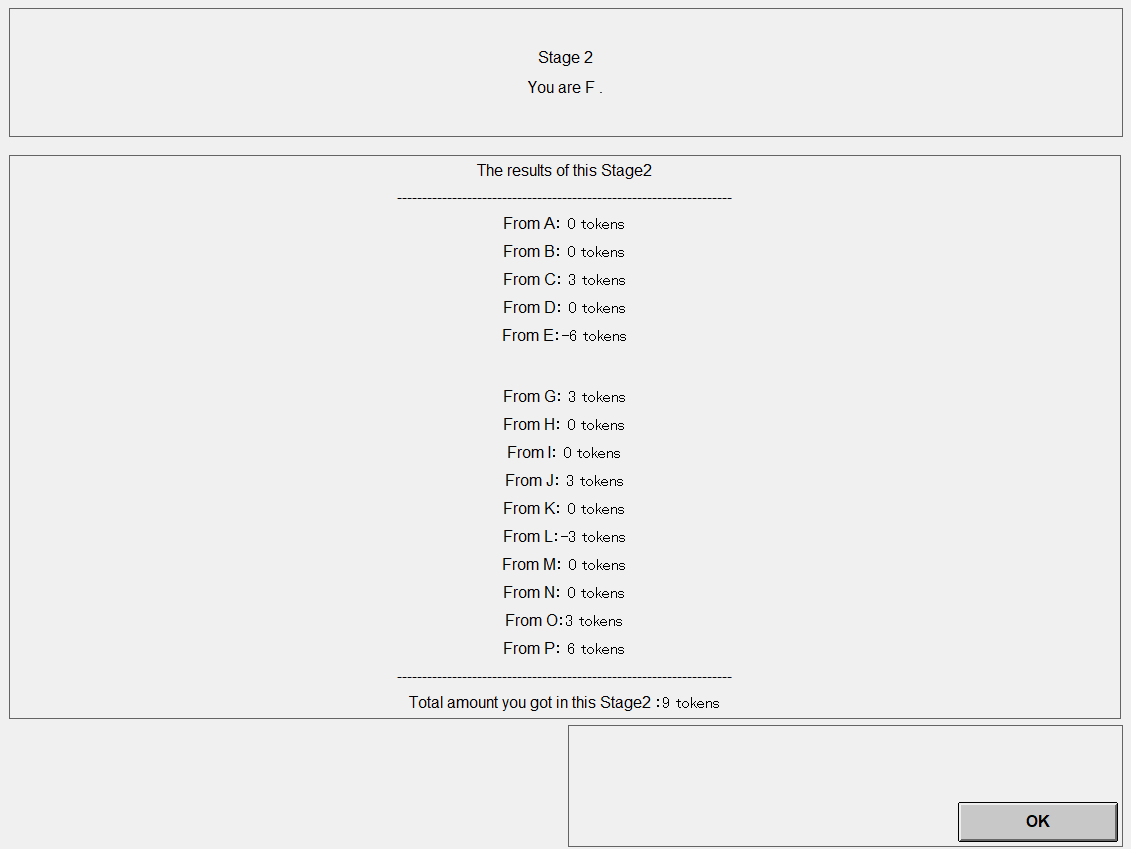


Screen shot of computer display when showing feedback after stage 2

(Global0.1 condition)
